# Association of prior beta-blocker treatment and outcome in patients with out-of-hospital cardiac arrest

**DOI:** 10.1007/s00392-026-02968-6

**Published:** 2026-06-22

**Authors:** Fardin Hamidi, Fabian Muhler, Gisela Skopp, Jonas Rusnak, Claudius Speer, Guido Michels, Norbert Frey, Thomas A. Zelniker, Michael R. Preusch

**Affiliations:** 1 Department of Cardiology, Angiology and Intensive Medicine CBF, Deutsches Herzzentrum der Charité - Universitätsmedizin Berlin, Berlin, Germany; 2 Division of Cardiology, Department of Medicine, Medical University of Vienna, Vienna, Austria; 3 Department of Cardiology, Angiology, and Pneumology, University Hospital Heidelberg, Im Neuenheimer Feld 410, 69120 Heidelberg, Germany; 4 Forensic Toxicology Center GmbH, Munich, Germany; 5 DZHK (German Centre for Cardiovascular Research), Partner Site Heidelberg/Mannheim, Heidelberg, Germany; 6 Department of Emergency Medicine, Hospital of the Barmherzige Brüder Trier, Trier, Germany

**Keywords:** Out-of-hospital cardiac arrest, Beta-blockers, Plasma drug concentrations, In-hospital mortality, Defibrillation

## Abstract

**Background:**

Out-of-hospital cardiac arrest (OHCA) is associated with poor outcomes and presents a considerable treatment challenge. Here, we investigated the association of prior beta-blocker treatment with outcomes in patients with OHCA.

**Methods:**

We enrolled consecutive OHCA patients with return of spontaneous circulation (ROSC). Beta-blocker plasma concentrations were measured upon admission and analyzed in relation to the number of defibrillations required to achieve ROSC, initial cardiac rhythm and in-hospital mortality.

**Results:**

Among 207 enrolled patients (median age 68 years, 71% male, 45% shockable rhythm), 65 (31.4%) had detectable beta-blocker plasma concentrations. Detectable beta-blocker concentrations were not associated with the likelihood of an initial shockable rhythm (adjusted odds ratio [OR] 0.99 [95% confidence interval [CI], 0.52–1.91]; *P* = 0.99), number of defibrillations (adjusted incidence rate ratio 0.93 [95% CI 0.63–1.37], *P* = 0.72) or doses of epinephrine (adjusted mean 7.4 vs. 6.7 mg, *P* = 0.29) and amiodarone (adjusted mean 99.3 vs. 93.9 mg, *P* = 0.83) administered to achieve ROSC. When analyzed as a continuous variable, beta-blocker concentration on admission was not associated with in-hospital death (adjusted OR 0.85 [95% CI 0.67–1.08]; *P* = 0.18). However, patients with plasma concentrations above the median had lower odds of death compared to those with undetectable levels or those below the median (adjusted OR 0.36 [95% CI 0.13–0.96]; *P* = 0.042).

**Conclusion:**

In OHCA patients with ROSC, detectable beta-blocker plasma concentrations on admission were not associated with defibrillation requirements or initial cardiac rhythm. While any detectable beta-blocker level was not associated with overall mortality, levels above the median were associated with lower odds of death in this cohort. These results are hypothesis-generating and warrant confirmation in larger independent cohorts.

**Graphical Abstract:**

**Figure:**
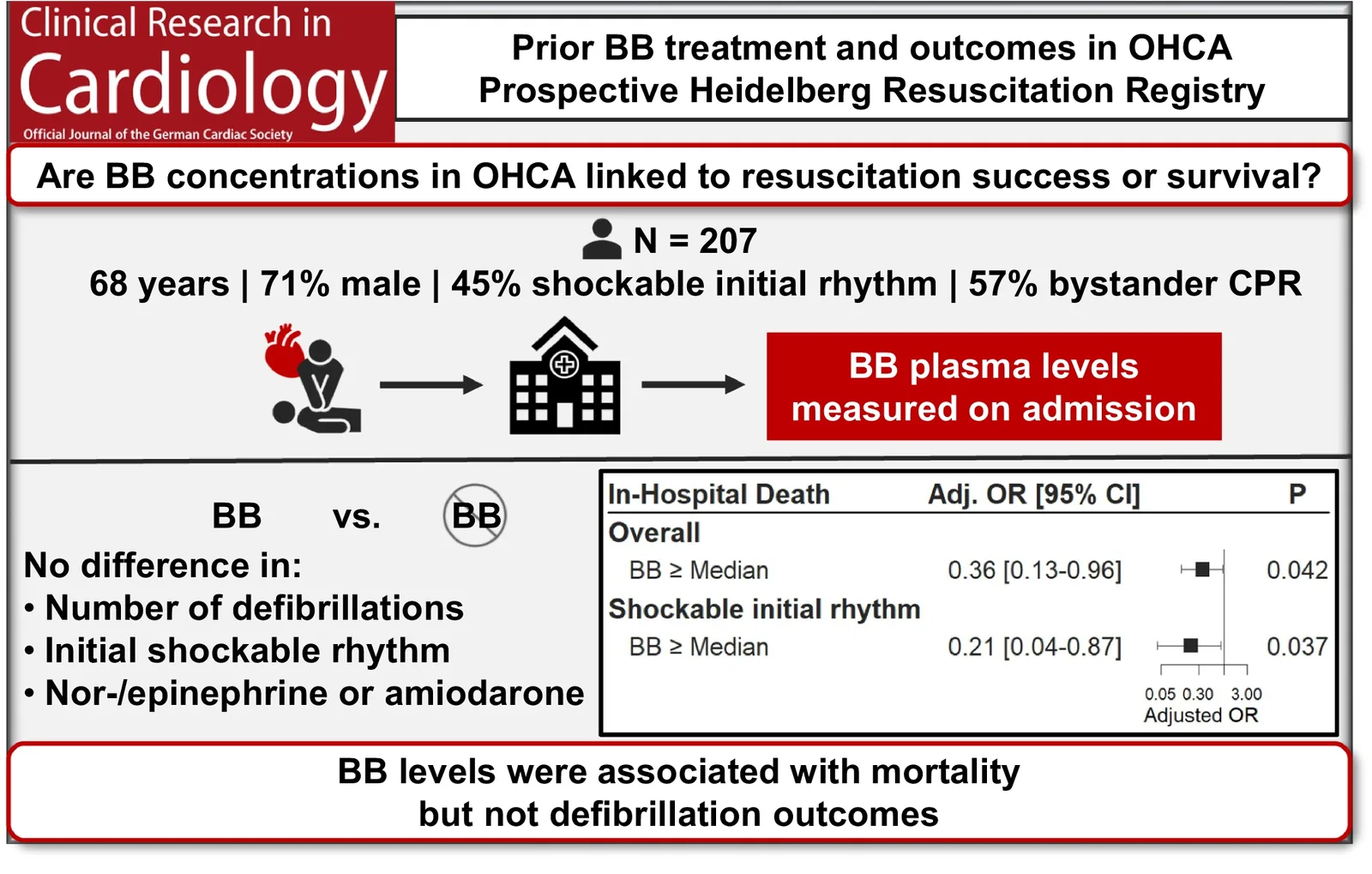

**Supplementary Information:**

The online version contains supplementary material available at https://doi.org/10.1007/s00392-026-02968-6.

## Background

Out-of-hospital cardiac arrest (OHCA) is associated with poor outcomes and remains a considerable challenge in clinical practice [1]. Current guidelines recommend an early defibrillation strategy with sequential shocks and a rapid three-shock strategy (escalating or fixed energy) that may improve termination rates for ventricular fibrillation (VF)/pulseless ventricular tachycardia (pVT) [2]. Although early defibrillation remains the cornerstone of therapy, some patients remain in arrhythmia despite repeated shocks and it offers no benefit to patients with non-shockable cardiac rhythms, underscoring the need for adjunctive pharmacologic interventions.

Despite the rapidly evolving field of cardiovascular pharmacotherapy, evidence considering the pharmacotherapy during cardiac arrest is limited [1]. Although epinephrine is recommended for treating cardiac arrest due to its activation of α receptors in vascular smooth muscle cells, which increases aortic diastolic pressure and coronary perfusion pressure, its concurrent stimulation of β1 receptors in cardiac muscle cells can induce ventricular arrhythmias [3–5]. Evidence from both animal experiments and retrospective human studies suggests that β receptor blockade (BB) reduces the risk of VF and may improve both return of spontaneous circulation (ROSC) and overall survival outcomes [6–8].

Therefore, we aimed to investigate the association of BB plasma levels with defibrillation outcomes and survival in patients with OHCA.

## Materials and methods

Patients after successful ROSC following a non-traumatic OHCA admitted to the intensive care unit of the University Hospital of Heidelberg between 01/2014 and 12/2016 were included in a consecutive fashion. The Heidelberg Resuscitation Registry has been described previously [9–11]. Patients were treated according to local critical care standard procedures, adhering to current guidelines. This included biphasic defibrillation with guideline-recommended shock energy, electrode placement predominantly in the anterior-lateral position and target temperature management (33 °C [± 1]). Data on patient demographics and event-specific characteristics were collected according to the Utstein-style criteria. Left-ventricular ejection function (LVEF) was derived from echocardiographic exams or visual assessment during ventriculographic exams within 3 days after admission [12]. Refractory VF/VT was defined as a VF/VT rhythm persisting despite three shocks at the first three rhythm checks [13]. Epinephrine administration was estimated based on resuscitation duration, assuming a dosage of 1 mg every 4 min. The study protocol complies with the declaration of Helsinki and was approved by the local ethics committee (S388-2011).

### Laboratory analysis and beta-blocker concentrations

Laboratory measures were conducted immediately upon admission, with plasma samples isolated within 60 min and stored at − 80 °C. Quantitative analysis of atenolol, metoprolol, bisoprolol, nebivolol, and carvedilol was performed using an Agilent 1290 Infinity LC system (Agilent Technologies, Waldbronn, Germany) coupled to an AB Sciex QTRAP 6500 MS (AB Sciex, Darmstadt, Germany), following protein precipitation and calibration procedures. The detailed method is described in the Supplementary Methods.

### Definition of endpoints

The primary outcome of this study was death before hospital discharge.

Secondary endpoints included initial cardiac rhythm, number of defibrillations required to achieve ROSC and amiodarone/epinephrine demand during resuscitation efforts.

### Statistical analysis

Categorical data are reported as counts and percentages, while continuous variables are represented as medians and interquartile ranges (IQRs). Chi-square tests were used for the analysis of discrete data, whereas the Mann–Whitney U test was used for comparing continuous data. A logistic regression model adjusted for age, sex, and bystander cardiopulmonary resuscitation (CPR) was used to examine the relationship between detectable BB concentrations and the likelihood of an initial shockable rhythm or refractory shockable rhythms. Negative binomial regression, adjusted for age, sex, bystander CPR and shockable initial rhythm, was used to examine the association between detectable or log-transformed BB levels and the number of defibrillations required for ROSC. Additionally, we used logistic regression models adjusted for age, sex, bystander CPR, shockable initial rhythm, eGFR and lactate on admission to assess the relationship between log-transformed BB levels and outcomes of interest. Additionally, subgroup analyses were conducted for age (< 68 versus > 68 years), sex, diabetes mellitus, hypertension, atrial fibrillation, renal impairment (eGFR < 60 ml/min versus ≥ 60 ml/min), shockable initial rhythm, lactate on admission, ST-segment Elevation Myocardial Infarction (STEMI) as the index event and prior history of myocardial infarction. Moreover, outcomes were also assessed according to the underlying cause of OHCA and first assessment of LVEF within 3 days of admission To analyze the relationship between detectable BB levels and the use of nor-/epinephrine and amiodarone, we applied linear regression with identical adjustment. Furthermore, we applied restricted cubic splines to assess non-linear relationships between log-transformed BB levels and time to ROSC. Linear regression models were employed to evaluate the association between emergency call time and BB concentrations. Two-sided P-values < 0.05 were considered to indicate statistical significance. All analyses were performed using R (Version 4.0.4).

## Results

### Study population

Out of 207 enrolled individuals (median age: 68 years; 71% male; 45% shockable initial rhythm; 57% bystander CPR; 87% presumed cardiovascular etiology of cardiac arrest; 39% documented BB prescription), 65 (31.4%) patients had detectable BB plasma levels on admission with a median plasma concentration of 19.60 ng/ml (IQR 5.70–49.60). The most frequently identified BB was bisoprolol (25%), followed by metoprolol (22%) and carvedilol (15%). In 15 (23%) cases with detectable BB concentrations, the specific agent could not be determined. Interestingly, among patients without detectable BB concentrations, 21% had a BB documented as part of their home medication. Information on home medication was unavailable in 19% of patients with detectable BB concentrations and in 25% of patients without detectable BB concentrations.

Individuals with detectable BB levels were more likely to be older (74 years vs. 66 years; *P* < 0.001), female (38% vs. 24%; *P* = 0.032), obese (48% vs. 22%; *P* < 0.001), have hypertension (86% vs. 67%; *P* = 0.006) and dyslipidemia (51% vs. 34%; *P* = 0.020, Table 1). Moreover, patients with detectable BB concentrations had lower eGFR (48 vs. 60 mL/min/1.73m^2^, *P* = 0.039) and higher C-reactive protein levels (10 vs 5 mg/L, *P* = 0.045) upon admission. There was no significant difference in the frequency of initially shockable rhythms (VF/VT) (42% vs. 47%; *P* = 0.45) or refractory VF/VT (18% vs. 18%, *P* = 0.90) between patients with and without detectable BB plasma levels. Within 3 days of hospital admission, both the distribution of LVEF categories (*P* = 0.74) and the prevalence of severely reduced LVEF (< 30%) (28% vs. 33%, *P* = 0.53) were similar between the two groups. The proportion of patients who had acute coronary syndrome (ACS) or ischemic cardiomyopathy as cause of the OHCA was similar between patients with detectable BB concentrations and those without (43 vs. 49%, *P* = 0.41). A numerically greater number of patients with detectable BB concentrations had nonischemic cardiomyopathy (including dilated, hypertrophic or takotsubo cardiomyopathy) (9% vs 3%, *P* = 0.075) or primary arrhythmia (11% vs. 4%, *P* = 0.053) as cause of cardiac arrest. Table 2 shows the baseline characteristics of patients with and without detectable BB concentrations stratified by initial shockable rhythm.

**Table 1:** Baseline characteristics of the study population stratified by presence of detected adrenergic β receptor blockade concentrations

	Overall *N* = 207	No BB detected *N* = 142	BB detected *N* = 65	*p*-value
**Age**	68 (57, 77)	66 (52, 76)	74 (66, 78)	** < 0.001**
**Male sex**	148 (71%)	108 (76%)	40 (62%)	**0.032**
**Diabetes mellitus**	61 (30%)	36 (26%)	25 (40%)	**0.045**
**Obesity**	61 (30%)	31 (22%)	30 (48%)	** < 0.001**
**Hypertension**	148 (73%)	94 (67%)	54 (86%)	**0.006**
**History of coronary artery disease**	80 (39%)	53 (38%)	27 (43%)	0.50
**Prior myocardial infarction**	59 (29%)	38 (27%)	21 (33%)	0.37
**Smoker**	58 (29%)	39 (28%)	19 (30%)	0.74
**Atrial fibrillation**	31 (15%)	23 (16%)	8 (12%)	0.47
**Dyslipidemia**	79 (39%)	47 (34%)	32 (51%)	**0.020**
**Biomarkers on admission**				
** Creatinine (mg/dL)**	1.27 (0.99, 1.61)	1.24 (0.99, 1.59)	1.32 (1.00, 1.63)	0.42
** eGFR (ml/min/1.72m**^2^)	56 (39, 74)	60 (42, 77)	48 (36, 65)	**0.039**
** HsTnT (ng/L)**	130 (57, 441)	144 (66, 483)	110 (41, 308)	0.17
** C-reactive protein (mg/L)**	6 (2, 23)	5 (2, 21)	10 (2, 35)	**0.045**
** Lactate (mg/dL)**	53 (18, 100)	51 (22, 96)	54 (2, 108)	0.98
** ST-segment elevation**	42 (20%)	32 (23%)	10 (15%)	0.24
** Cardiovascular cause of arrest**	181 (87%)	123 (87%)	58 (89%)	0.60
**Causes of cardiac arrest**				
** ACS or ischemic CMP**	98 (47%)	70 (49%)	28 (43%)	0.41
** Nonischemic CMP**	10 (4.8%)	4 (2.8%)	6 (9.2%)	0.075
** IMPS**	4 (1.9%)	4 (2.8%)	0 (0%)	0.31
** Primary arrhythmia**	12 (5.8%)	5 (3.5%)	7 (11%)	0.053
** Pulmonary embolism**	11 (5.3%)	8 (5.6%)	3 (4.6%)	1.00
** Unspecified cardiovascular cause**	46 (22%)	32 (23%)	14 (22%)	0.87
**Non-cardiac cause of arrest**	26 (13%)	19 (13%)	7 (11%)	0.60
**LVEF**				
** > 52%**	53 (28%)	38 (29%)	15 (25%)	0.57
** 41–51%**	31 (16%)	20 (15%)	11 (18%)	0.59
** 30–40%**	47 (25%)	30 (23%)	17 (28%)	0.42
** < 30%**	60 (31%)	43 (33%)	17 (28%)	0.53
** Bystander CPR**	117 (57%)	83 (58%)	34 (52%)	0.41
** pH upon admission**	7.16 (6.99, 7.28)	7.16 (6.99, 7.28)	7.13 (7.01, 7.25)	0.76
** Shockable initial rhythm**	94 (45%)	67 (47%)	27 (42%)	0.45
** Refractory VF/VT**	37 (18%)	25 (18%)	12 (18%)	0.90
** Time to ROSC**	25 (18, 40)	27 (20, 40)	22 (15, 35)	0.14
**Number of defibrillations**				
** 0**	93 (45%)	60 (43%)	33 (51%)	0.29
** 1–2**	52 (25%)	38 (27%)	14 (22%)	0.39
** ≥ 3**	60 (29%)	42 (30%)	18 (28%)	0.74
** > 0**	112 (55%)	80 (57%)	32 (49%)	0.29
**Amiodaron administration during CPR (mg)**				
** 0**	140 (71%)	92 (69%)	48 (75%)	0.36
** 150**	7 (3.5%)	6 (4.5%)	1 (1.6%)	0.43
** 300**	29 (15%)	18 (13%)	11 (17%)	0.48
** > 300**	22 (11%)	18 (13%)	4 (6.3%)	0.13
** > 0**	58 (29%)	42 (31%)	16 (25%)	0.36

**Table 2:** Comparison of patients with an initially shockable and non-shockable rhythm based on detected adrenergic β receptor blockade

	Non-shockable rhythm			Shockable Rhythm		
	No BB detected *N* = 75	BB detected *N* = 38	*p*-value	No BB detected *N* = 67	BB detected *N* = 27	*p*-value
**Age**	68 (51, 78)	76 (69, 82)	**0.006**	64 (52, 73)	70 (61, 76)	0.10
**Male sex**	49 (65%)	21 (55%)	0.30	59 (88%)	19 (70%)	0.065
**Diabetes mellitus**	23 (31%)	18 (50%)	0.054	13 (20%)	7 (26%)	0.51
**Obesity**	18 (24%)	19 (53%)	**0.003**	13 (20%)	11 (41%)	**0.035**
**Hypertension**	47 (64%)	31 (86%)	**0.014**	47 (71%)	23 (85%)	0.16
**History of coronary artery disease**	25 (34%)	15 (42%)	0.42	28 (42%)	12 (44%)	0.86
**Prior myocardial infarction**	16 (22%)	10 (28%)	0.48	22 (33%)	11 (41%)	0.50
**Smoker**	19 (26%)	8 (22%)	0.69	20 (30%)	11 (41%)	0.33
**Atrial fibrillation**	14 (19%)	5 (13%)	0.46	9 (13%)	3 (11%)	1.00
**Hyperlipoproteinemia**	25 (34%)	20 (56%)	**0.029**	22 (33%)	12 (44%)	0.31
**Biomarkers on admission**						
** Creatinine (mg/dL)**	1.34 (0.99, 1.83)	1.36 (1.06, 1.79)	0.90	1.18 (0.99, 1.34)	1.25 (0.94, 1.55)	0.39
** eGFR (ml/min/1.72m**^**2**^**)**	49 (31, 77)	47 (35, 62)	0.39	66 (52, 77)	57 (41, 73)	0.096
** HsTnT (ng/L)**	127 (62, 354)	98 (50, 221)	0.26	193 (67, 600)	145 (38, 573)	0.52
** C-reactive protein (mg/L)**	10 (2, 30)	17 (3, 46)	0.22	2 (2, 12)	4 (2, 17)	0.091
** Lactate (mg/dL)**	68 (25, 102)	65 (30, 116)	0.57	47 (20, 72)	38 (1, 64)	0.32
** ST-segment elevation**	11 (15%)	2 (5.3%)	0.21	21 (31%)	8 (30%)	0.87
** Cardiovascular cause of arrest**	56 (75%)	32 (84%)	0.25	67 (100%)	26 (96%)	0.29
**Cause of arrest**						
** ACS or ischemic CMP**	19 (25%)	14 (37%)	0.20	51 (76%)	14 (52%)	0.021
** Nonischemic CMP**	2 (2.7%)	2 (5.3%)	0.60	2 (3.0%)	4 (15%)	0.055
** IMPS**	1 (1.3%)	0 (0%)	1.00	3 (4.5%)	0 (0%)	0.55
** Primary arrhythmia**	0 (0%)	1 (2.6%)	0.34	5 (7.5%)	6 (22%)	0.072
** Pulmonary embolism**	7 (9.3%)	3 (7.9%)	1.00	1 (1.5%)	0 (0%)	1.00
** Unspecified cardiovascular cause**	27 (36%)	12 (32%)	0.64	5 (7.5%)	2 (7.4%)	1.00
** Non-cardiac cause of arrest**	19 (25%)	6 (16%)	0.25	0 (0%)	1 (3.7%)	0.29
** Bystander CPR**	36 (48%)	17 (45%)	0.74	47 (70%)	17 (63%)	0.50
** pH upon admission**	7.09 (6.89, 7.24)	7.08 (6.95, 7.22)	0.87	7.24 (7.08, 7.32)	7.21 (7.09, 7.35)	0.96
** Time to ROSC**	25 (20, 40)	24 (15, 35)	0.52	28 (18, 41)	22 (16, 30)	0.13
**Number of defibrillations**						
** 0**	57 (76%)	33 (87%)	0.18	3 (4.6%)	0 (0%)	0.55
** 1–2**	10 (13%)	4 (11%)	0.77	28 (43%)	10 (37%)	0.59
** ≥ 3**	8 (11%)	1 (2.6%)	0.27	34 (52%)	17 (63%)	0.35
** > 0**	18 (24%)	5 (13%)	0.18	62 (95%)	27 (100%)	0.55
**Amiodaron administration during CPR (mg)**						
** 0**	62 (86%)	34 (92%)	0.54	30 (48%)	14 (52%)	0.76
** 150**	2 (2.8%)	1 (2.7%)	1.00	4 (6.5%)	0 (0%)	0.31
** 300**	4 (5.6%)	2 (5.4%)	1.00	14 (23%)	9 (33%)	0.29
** > 300**	4 (5.6%)	0 (0%)	0.30	14 (23%)	4 (15%)	0.40
** > 0**	10 (14%)	3 (8.1%)	0.54	32 (52%)	13 (48%)	0.76

Since BBs are typically taken in the morning, we hypothesized that patients who experienced OHCA earlier in the day might have higher plasma BB concentrations at admission. To explore this, we used the emergency call time as a surrogate marker for the time of OHCA and compared BB concentrations between patients with morning (before 12:00) versus later arrests. However, we found no significant difference in BB concentrations or detectability based on the time of day (both *P* > 0.05).

### Beta-blocker concentrations and defibrillation outcomes

**Fig. 1:**
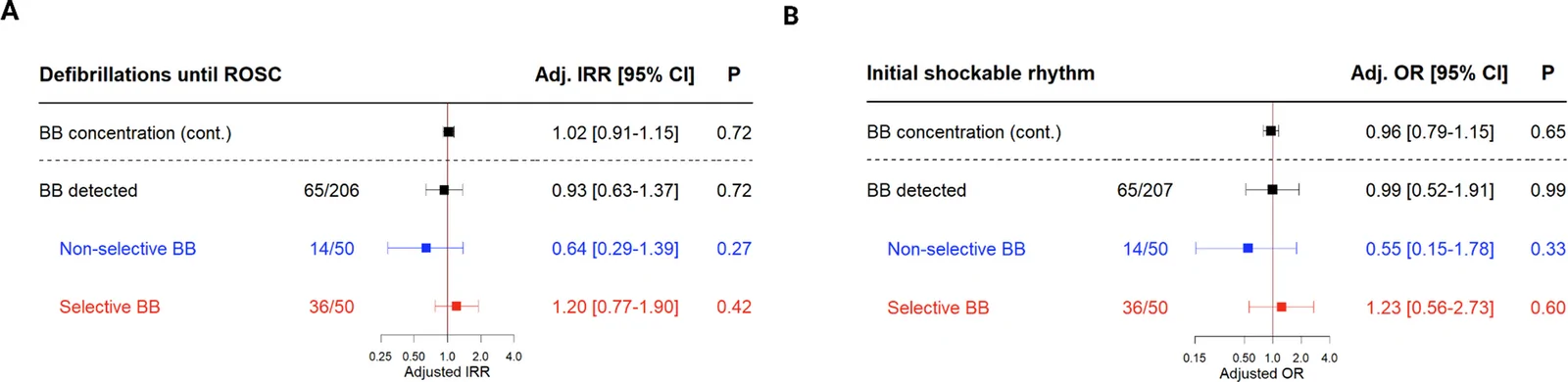
Association of BB concentrations, overall and stratified by β1-receptor selectivity, with (**A**) defibrillations until ROSC and (**B**) likelihood of an initial shockable rhythm. Continuous BB concentrations were log-transformed. The type of BB could not be determined in 15 patients. The number of defibrillations until ROSC is unknown in 1 patient. Models were adjusted for age, sex, bystander CPR and shockable initial rhythm when not used as an endpoint

Overall, we observed no difference in the number of defibrillations needed to achieve ROSC between patients with and without detectable BB concentrations (adjusted (adj.) incidence rate ratio [IRR] 0.93 [95% confidence interval [CI] 0.63–1.37], *P* = 0.72, Fig. 1A). This finding remained consistent regardless of the β1-selectivity of the measured concentrations (*P* > 0.05 for both). Moreover, detectable BB concentrations (adj. OR 1.20 [95% CI 0.52–2.66], *P* = 0.66)—whether β1-selective (adj. OR 2.05 [95% CI 0.82–4.97], *P* = 0.11) or nonselective BBs (adj. OR 0.34 [95% CI 0.02–1.90], *P* = 0.31) – were not significantly associated with refractory VF/VT compared to patients without detectable BB concentrations.

Similarly, patients with detectable BB concentrations -whether selective or non-selective (both *P* > 0.10)—did not have higher odds of presenting with an initial shockable rhythm after multivariable adjustment compared to those without detectable BB concentrations (adj. odds ratio [OR] 0.99 [95% CI 0.52–1.91], *P* = 0.99, Fig. 1B).

There was also no evidence of a linear or non-linear relationship between BB levels and the likelihood of an initial shockable rhythm, refractory VF/VT, the number of defibrillations for ROSC and time to ROSC (*P* > 0.05, Supplemental Fig. 1).

### Association of beta-blocker plasma levels and pharmacological interventions during OHCA

Detectable BB concentrations were not associated with higher doses of epinephrine (Adj. Mean 7.4 mg vs. 6.7 mg, *P* = 0.29) or amiodarone (Adj. Mean 99.3 mg vs. 93.9 mg, *P* = 0.83) during resuscitation, nor in the amount of norepinephrine used within the first 24 h following emergency medical service contact (Adj. Mean 27.5 mg vs. 28.0 mg, *P* = 0.89). Treatment with amiodarone resulted in similar defibrillation rates for achieving ROSC in patients with and without detectable BB plasma concentrations (*P* value for interaction = 0.97). Likewise, epinephrine administration did not modify the number of delivered defibrillations between these groups (*P* value for interaction = 0.97).

### Beta-blocker plasma levels and in-hospital mortality

Of 207 patients, 133 (64%) died during the in-patient stay. Mortality rates were 63% (90/142) in those without detectable BB concentrations and 66% (43/65) in those with detectable levels (adj. OR 0.74 [95% CI 0.33–1.66], *P* = 0.47). Neither detectable β1-selective BB concentrations (adj. OR 0.67 [95% CI 0.26–1.74], *P* = 0.41) nor non-selective BB levels (adj. OR 0.94 [95% CI 0.21–5.33], *P* = 0.94) were independently associated with death. These findings were consistent regardless of the initial cardiac rhythm (P value for interaction = 0.23). When analyzed as a log-scaled continuous variable, BB plasma concentration on admission was not associated with in-hospital death (adjusted OR 0.85 [95% CI, 0.67–1.08], *P* = 0.47). However, patients with BB levels above the median had significantly lower odds of in-hospital death than those below the median (adj. OR 0.25 [95% CI 0.06–0.94], *P* = 0.045) and showed a trend toward lower odds compared to patients without detectable BB (adj. OR 0.38 [95% CI 0.14–1.06], *P* = 0.065; Supplemental Fig. 2).

Furthermore, we observed lower odds of in-hospital death (adj. OR 0.01 [95% CI 0.00–0.26], *P* = 0.018; P for interaction = 0.026) in patients with refractory VF/VT with detectable BB concentrations above or equal to the median.

**Fig. 2:**
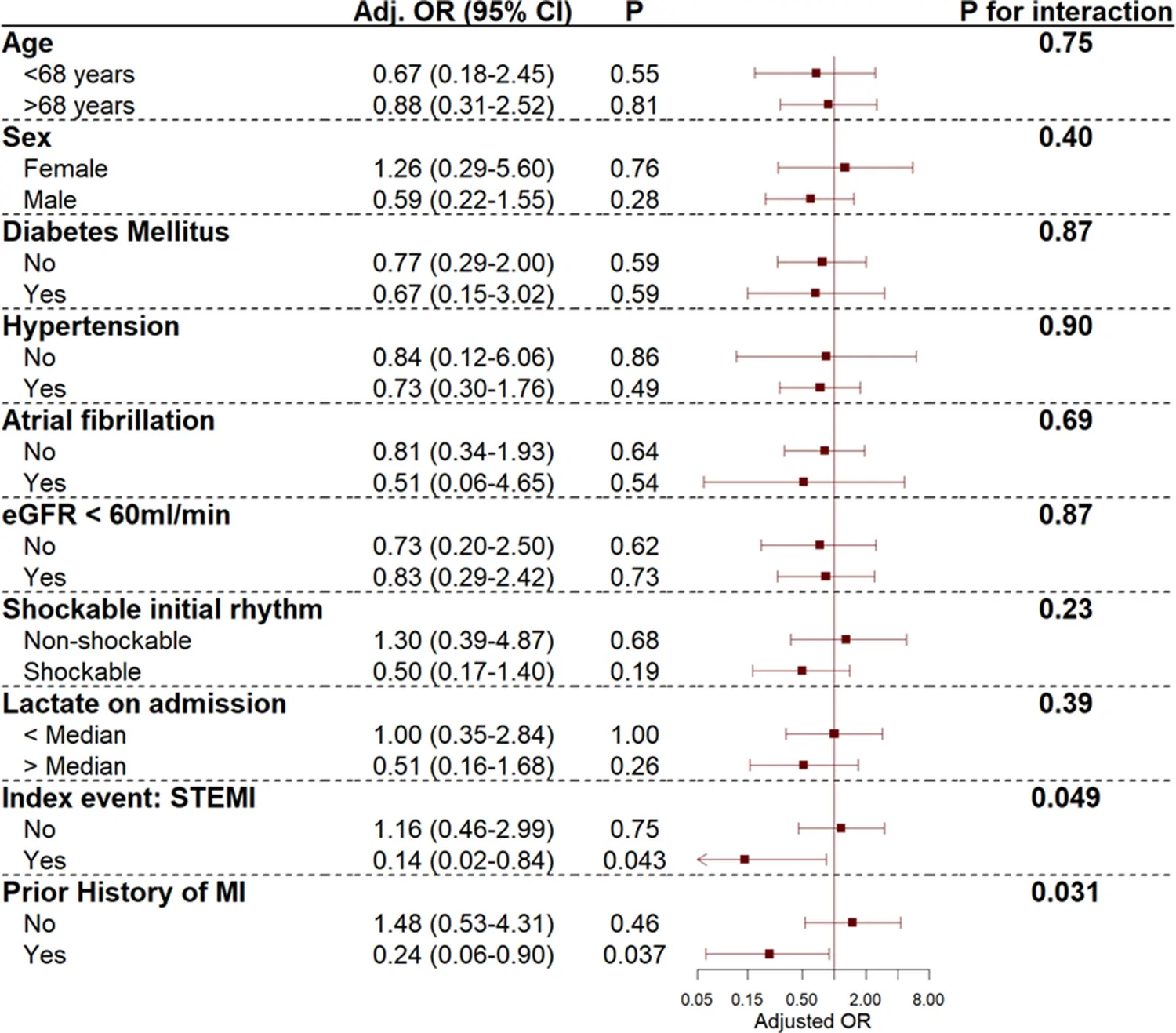
Association of BB concentrations (detectable vs. not-detectable) and in-hospital death among different clinical subgroups

Subgroup analyses revealed that detectable BB concentrations on admission were associated with reduced in-hospital mortality in patients with STEMI as the index event (adj. OR 0.14 [95% CI 0.02–0.84]; *P* = 0.043; P for interaction = 0.049), whereas no association was observed in patients without STEMI, including those without myocardial infarction. A similar pattern was observed, in patients with prior myocardial infarction (adj. OR 0.24 [95% CI 0.06–0.90]; *P* = 0.037) but not in those without (adj. OR 1.48 [95% CI 0.53–4.31]; *P* = 0.46; P value for interaction = 0.031, Fig. 2). Using a median-based cutoff, this interaction persisted and was even more pronounced. BB concentrations above the median were strongly associated with lower odds of death in patients with a prior history of myocardial infarction compared to those without detectable or below median BB concentrations (adjusted OR 0.06 [95% CI, 0.01–0.28]; *P* < 0.001; P for interaction = 0.005), whereas no significant interaction was observed in the other analyzed subgroups. Likewise, no significant association between detectable BB concentrations and in-hospital mortality was observed across the remaining subgroups, including age (< 68 vs. ≥ 68 years), sex, diabetes mellitus, hypertension, atrial fibrillation, eGFR (< 60 vs. ≥ 60 ml/min), initial rhythm (shockable vs. non-shockable), and admission lactate (< median vs. ≥ median, all *P*-values > 0.05). Moreover, detectable BB concentrations were associated with lower in-hospital mortality among patients with an ischemic cause of cardiac arrest (adj. OR 0.31 [95% CI 0.09–0.95]; *P* = 0.048), whereas no significant association was observed in non-ischemic cardiovascular etiologies (adj. OR 1.45 [95% CI 0.34–6.71]; *P* = 0.62).

Lastly, detectable BB concentrations above the median were associated with lower mortality in patients with LVEF ≤ 40% (adj. OR 0.09 [95% CI 0.01–0.49]; *P* = 0.008, Supplemental Table 1). Additionally, among patients with an ischemic cause of cardiac arrest, above-median BB plasma levels were associated with lower odds of death irrespective of LVEF.

## Discussion

To our knowledge, this is the first study to explore the association between pre-arrest BB therapy verified by plasma concentrations at the time of arrest and outcomes in OHCA patients. Despite the established benefits of chronic BB therapy in preventing and treating cardiac arrhythmia, detectable BB concentrations at admission were not associated with improved defibrillation success or a higher likelihood of an initial shockable rhythm. Interestingly, however, among those who presented with an initial shockable rhythm/refractory VF/VT, higher beta-blocker plasma concentrations were associated with better in-hospital survival compared to patients without detectable BB concentrations. The association between prior BB therapy and OHCA outcomes has been investigated only in a few studies, all of which identified chronic BB therapy based on electronic medical records or patient-reported information [14–17]. However, in acute settings, these sources are often unreliable, incomplete or outdated. This is particularly relevant in acute care, where timely decisions depend on a patient's current pharmacological status rather than potentially inaccurate historical records. Our study is unique in quantifying BB plasma concentrations on admission rather than relying on prescription records or patient history. Notably, although 39% of patients were on documented BB therapy, many had no measurable plasma concentrations, suggesting potential non-compliance, altered drug metabolism or other influencing factors. This discrepancy in historic documentation and acute patient status prompts the question of the requirement and necessity of routine, real-time pharmacological profiling—especially for medications with significant hemodynamic effects—and their role in intensive care for future research. Since current guidelines do not recommend BB in OHCA patients and such treatment would have been documented in pre-hospital records had it occurred, it is reasonable to assume they did not receive pre-hospital BB treatment during resuscitation, allowing for a precise evaluation of the effects of chronic beta-blockade at the time of OHCA in this study. Plasma BB levels indicate recent exposure but cannot reliably quantify dose due to variability in drug type, timing of last dose, adherence and renal/hepatic function between patients. CYP3A4- and CYP2D6(18)-dependent metabolism, especially for bisoprolol, carvedilol, and metoprolol, introduces additional variability, particularly in patients with unknown metabolizer phenotype [18]. In addition, BB-induced hemodynamic effects do not correlate linearly with plasma concentrations, suggesting inter-individual variability in response mechanisms. While most data on these limitations derive from stable clinical settings [19, 20], variability is likely intensified exponentially in OHCA. Still, detectable plasma levels remain a direct marker of drug presence and may offer a more accurate representation of exposure than medical records or self-report.

In our OHCA cohort, nearly one-third of patients had detectable BB levels, yet defibrillation success and the incidence of an initial shockable rhythm did not differ significantly. In theory, chronic BB therapy could blunt the adverse beta-adrenergic effects of repeated epinephrine administration during CPR. While BB therapy may reduce adrenergic drive and myocardial excitability, our findings suggest these effects do not meaningfully impact immediate defibrillation outcomes or escalate medication requirements for ROSC, as both groups received similar doses of epinephrine and amiodarone during resuscitation. The detection of BB concentrations in plasma most likely reflects underlying cardiovascular disease burden and treatment context rather than an independent causal effect. This is supported by the higher prevalence of cardiovascular comorbidities among patients with measurable BB concentrations. However, the observed association between higher BB concentrations and lower in-hospital mortality in selected subgroups, particularly in patients with ischemic etiologies and prior MI, suggests that BB exposure may not be a purely passive marker. However, these findings remain exploratory and should be considered hypothesis-generating. These findings may reflect differences in treatment adherence, disease stability or other unmeasured factors. Importantly, given the observational design and the potential for residual confounding, a causal relationship between BB concentrations and improved outcomes cannot be established and requires validation in larger independent cohorts. Taken together, these findingssuggest the interpretation of BB concentrations primarily as a marker of underlying disease and treatment status, with a possible additional association in specific clinical contexts.

Consistent with this notion, in-hospital mortality also differed between patients with above-median BB plasma concentrations and those with below median or undetectable concentrations. While BB plasma concentration is not directly equivalent to prescribed dose, this observation is partially consistent with prior literature where a prescription history of high-dose BB therapy seemed to be associated with more favorable short-term and long-term survival when compared to no BB therapy [15]. In contrast, other studies [14, 16] that did not stratify by low and high-dose BB therapy showed no significant mortality differences. Higher BB plasma concentrations may be a marker of better adherence to home medications, superior management of cardiovascular risk factors or a preserved pharmacokinetic state in the acute setting of OHCA, warranting further investigation.

### Limitations

We acknowledge several limitations: First, although we used mass spectrometry for BB detection, eliminating the risk of cross-reactions that can occur in immunoassays, we were unable to assess individual dose–response relationships for plasma BB levels. However, this limitation applies broadly to many pharmacokinetic studies. Second, while pharmacokinetic and pharmacodynamic profiling cannot be performed from a single sample, the study focused on measuring drug concentrations at the time of arrest, when they are most likely to influence clinical outcomes. Third, while we approximated the index event using emergency call times to explore the potential association of morning versus later BB intake, variability in plasma BB levels remains a potential confounder. Fourth, this prospective registry primarily included patients with ROSC upon arrival. Since ECLS and ECPR were not yet common practice at the time, patients without ROSC remain an important population for future investigation. Fifth, the study cohort was derived from patients enrolled between 2014 and 2016, which may limit the generalizability of our findings to contemporary OHCA populations. Sixth, epinephrine administration was estimated based on resuscitation duration. However, real-world intra-arrest drug administration can be highly variable and deviate from guideline-recommended intervals. Moreover, measures of medication adherence, timing of last BB-intake, prescribed dose and pharmacy refill records as well as detailed previous medical history were lacking.. In addition, longitudinal systematic data on LV-function were not available and single-time-point measurements of LV-function are susceptible to OHCA-induced alterations, such as reperfusion injury and myocardial stunning, which may limit interpretability. Lastly, although our analyses were adjusted for several clinically relevant variables, residual confounding cannot be excluded and given the exploratory and hypothesis‐generating nature of our study, no adjustments for multiple testing were performed.

## Conclusion

In this study, detectable BB plasma concentrations at the time of OHCA were not associated with increased defibrillation success or higher pharmacologic requirements during resuscitation. In this cohort BB plasma concentrations elevated above the median were associated with lower in-hospital mortality, particularly in patients with refractory shockable rhythms in exploratory analysis.

## Supplementary Information

Below is the link to the electronic supplementary material.

Supplementary file1 (DOCX 170 KB)

## Data Availability

The data underlying this article will be shared on reasonable request to the corresponding author.
