# Supplementary material for: Association of prior beta-blocker treatment and outcome in patients with out-of-hospital cardiac arrest
Source: Clin Res Cardiol. 2026 Jun 22;115(11):2038–47. doi: 10.1007/s00392-026-02968-6 (PMC13658337; doi:10.1007/s00392-026-02968-6)
Supplement: Supplementary file 1 [file 392_2026_2968_MOESM1_ESM.docx]

# Supplemental Figures

## Figure 1: Association between logarithmic BB levels and time to ROSC

Linear regression adjusted for age, sex, bystander CPR and shockable initial rhythm

## Figure 2: Association between no detected BB concentrations, BB concentrations below and BB concentrations at or above the median with In-hospital death.

# Supplemental Tables

## Supplemental Table 1. Association between in-hospital death and BB concentrations among LVEF-subgroups

|  | **Detectable vs. non-detectable BB concentrations** | | | | **BB above median (vs. ≤ median or non-detectable)** | | | |
| --- | --- | --- | --- | --- | --- | --- | --- | --- |
|  | **Subgroup** | **Adj. OR (95% CI)** | **P** | **P for interaction** | **Subgroup** | **Adj. OR (95% CI)** | **P** | **P for interaction** |
| Overall | LVEF > 40% | 1.02 [0.37-2.87] | 0.96 | 0.16 | LVEF > 40% | 0.63 [0.18-2.36] | 0.48 | 0.068 |
|  | LVEF ≤ 40% | 0.31 [0.08-1.23] | 0.097 |  | LVEF ≤ 40% | 0.09 [0.01-0.49] | 0.008 |  |
| Ischemic cause of OHCA | LVEF > 40% | 0.22 [0.04-1.12] | 0.083 | 0.93 | LVEF > 40% | 0.04 [0.00-0.36] | 0.006 | 0.38 |
|  | LVEF ≤ 40% | 0.25 [0.04-1.42] | 0.12 |  | LVEF ≤ 40% | 0.01 [0.00-0.17] | 0.010 |  |

# Supplementary Methods

Laboratory measures were undertaken immediately upon admission and processed in accordance with local laboratory standards. In addition, serum and plasma were isolated separately within 60 min of sample acquisition and stored at −80°C until measurement. Certified reference solutions (1mg/ml in acetonitrile or methanol) of atenolol and metoprolol were purchased from Merck (Darmstadt, Germany), and of bisoprolol, nebivolol and carvedilol from LGC Standards (Wesel, Germany), respectively. Deuterated 3,4-methylenedioxymethamphetamine (MDMA-d5) and 5-(p-methylphenyl)-5-phenylhydantoin (MPPH) which were used as internal standards were from Merck (Darmstadt, Germany). Plasma samples were prepared for liquid chromatography/tandem mass spectrometry following protein precipitation of 100 µl with 1,0 ml acetonitrile prior to addition of the internal standards. Prepared calibrators (calibration ranges: atenolol, metoprolol 100-1000 ng/ml; bisoprolol 10-100 ng/ml; nebivolol 5-50 ng/ml; carvedilol 25-250 ng/ml), controls (n=2, above the lowest and below the highest calibration standards, respectively) and samples were analyzed with an Agilent 1290 Infinity liquid chromatography system (Agilent Technologies, Waldbronn, Germany) coupled to a AB Sciex QTRAP 6500 triple quadrupole mass spectrometer (AB Sciex, Darmstadt, Germany). Samples were separated on a Zorbax Eclipse C8 column (150 mm x 4.6 mm, Agilent Technologies, Waldbronn, Germany). The electrospray ionization was set to positive, and two transitions were monitored for each analyte. Compound identification was confirmed by retention time and respective product ion ratios (± 20%). Data collection and processing was carried out by using Analyst (AB Sciex, Darmstadt, Germany), and quantitation was performed with linear regression using 7-point calibration curves. The lower limit of detection was by a quarter of the lowest calibrator, respectively.
